# Unmet need for presbyopia correction and its associated factors among school teachers in Hawassa city, South Ethiopia

**DOI:** 10.1186/s12886-020-01454-5

**Published:** 2020-05-07

**Authors:** Minychil Bantihun Munaw, Balcha Negese Kebede, Nebiyat Feleke Adimassu

**Affiliations:** 1 Department of Optometry, College of Medicine and Health Sciences, University of Gondar, P.O.Box: 196, Gondar, Ethiopia; 2 Department of Ophthalmology and Optometry, College of Medicine and Health Sciences, University of Hawassa, Hawassa, Ethiopia

**Keywords:** Unmet need for presbyopia correction, School teachers, Hawassa Ethiopia

## Abstract

**Background:**

Presbyopia is a decline in the amplitude of accommodation with the onset in the age range 40–45 years affecting near visual task performance. As the age of presbyopia onset coincides with productive age, it results in great productivity loss especially in those with high near visual demand like teachers. A maximum near vision potential is essential for teachers in ensuring the quality of education, as most of the students’ evaluations and scripts are assessed manually in Ethiopia. The prevalence of unmet need for presbyopia correction among school teachers ranges from 38.5–70.4% worldwide. Though presbyopia is a common ocular condition, there is limited evidence regarding the unmet need for presbyopia correction in Ethiopia as well as in Hawassa city. Therefore, this study aimed to determine the magnitude of unmet need for presbyopia correction and its associated factors among school teachers in Hawassa city, South Ethiopia**.**

**Methods:**

A school-based cross-sectional study was conducted among teachers older than 35 years. A simple random sampling technique was used to select participants using name lists as a sampling frame from 69 schools. The participants selected underwent for distance and near visual acuity test. Those with distance visual acuity of 6/12 or worse were refracted before near visual acuity test. Information on the spectacle use and associated factors was obtained using the interviewer-administered questionnaire. Data analysis was done using Statistical Package for Social Sciences software version 20. Binary logistic regression analysis was performed to assess association between independent variables and the unmet need for presbyopia correction.

**Results:**

A total of 459 study participants were included in the study with a response rate of 95.21%.The unmet need for presbyopia correction was 51.26% (95%CI: 46.7–55.6%). Female gender (AOR = 2.50; 95%CI: 1.51–4.15), age 36–45 (AOR = 4.12; 95%CI: 1.46–11.76), unaware of presbyopia (AOR = 2.36; 95%CI: 1.2–4.66) and self-rating of current vision as good (AOR = 3.5; 95%CI: 1.61–7.6) were factors significantly associated with the unmet need for presbyopia.

**Conclusions:**

The burden of unmet need for presbyopia correction is a moderate priority according to the World Health Organization for presbyopia correction services criteria. A school-based presbyopia awareness creation program is important to reduce this huge burden.

## Background

Presbyopia is a global problem affecting over a billion people older than 34 years worldwide [1, 2]. It is a progressive age-related loss in the amplitude of accommodation due to crystalline lens growth and changes in its elastic properties with the onset of less than 40 years of age in females and Africans [3–5]. The number of people with presbyopia who do not have spectacles was estimated to be 826 million in 2015 [2].

The symptoms of presbyopia are visual discomfort such as eye strain, headache, and difficulty to perform near visual tasks such as reading [3, 6]. Lack of clear near vision prevents teachers from regular reading, faces difficulty in checking students’ class work, marking exams, and doing manuscripts, especially where those activities are done manually like in Ethiopia. Therefore, the cumulative effect of uncorrected presbyopia on daily teaching activities has a great impact on the quality of education.

As a result of their long work experience, the older teachers have an invaluable role in ensuring the quality of education. However, presbyopia limits these teachers from contributing to full potential. Besides, presbyopia has a huge economic impact because its onset coincides with productive age [5]. From the literatures reviewed, the unmet need for presbyopia correction ranges from 4 to 96.8% [7–14] in the community and 38.5 to 70.4% for school teachers [3, 4, 12, 15] worldwide.

When setting the priority for presbyopia service provision, the World Health Organization sets recommendations. It sets as high priority if only less than one-third presbyopics had presbyopia correction, moderate if one- third to two-third presbyopics had presbyopia correction and low if more than two-third presbyopics had presbyopia correction [16].

Although the burden of presbyopia affects productivity, there are many people left uncorrected for several reasons. Being female, high cost of spectacles, illiteracy, and lack of awareness about where to access presbyopia correction, availability, and access to eye care services were barriers for use of presbyopia corrections [1, 15–18].

A proper estimate of the unmet need for presbyopia correction is essential for advocacy, planning, establishing refraction, and spectacle distribution for teachers at the government level. However, there is little information on the unmet need for presbyopia correction and its associated factors among school teachers in Ethiopia, particularly in Hawassa city. Hence, it is imperative to estimate the unmet need for presbyopia correction and identify factors that affect access to correction among school teachers.

## Methods

### Study population and study design

A school-based cross-sectional study was conducted from April 25 to May 30, 2019, to assess the unmet need for presbyopia correction among school teachers in Hawassa city. Hawassa is the capital city of Southern Nation, Nationalities and Peoples’ Regional State (SNNPR) which is located 276 Km from Addis Ababa, the capital. The urban population of Hawassa in the 2018 projection was 266,331. From these 137,316 are males and 129,015 are females. It has eight urban sub-city administration and 20 Kebeles (local administrative). A total number of 1569 teachers older than 35 years were teaching in 69 schools in urban kebeles of Hawassa city, of which 963 were teaching in public schools and 606 were teaching in private schools. There are two hospitals, one comprehensive specialized hospital, and one general hospital, three private specialized higher eye clinics, and one medium optometry clinic providing eye care services including refraction (spectacle correction).

### Sample size determination and sampling procedure

The sample size was calculated for the proportion of unmet need using a single population proportion formula and associated factors using EPI INFO 7 with the assumption of 95% confidence level and power 80%. The largest sample size was chosen to assure the representativeness of the sample.

The sample size for the proportion of unmet need = $$ \frac{z\raisebox{1ex}{$\alpha $}\!\left/ \!\raisebox{-1ex}{$2P\left(1-P\right)$}\right.}{d^2} $$ = $$ \frac{(1.9)^2(0.692)(0.308)\ }{(0.05)^2} $$ = ≈ 327, where p- 0.692 from a community-based study in Bahir Dar city, North West Ethiopia [17], level of significance (95%) and margin of error (d) = 0.05.

Being female was one of the factors frequently associated with the unmet need for presbyopia correction in our literature reviews that gives a larger sample size. Using unexposed to exposed ratio 1:1, OR 1.78, outcome among unexposed (i.e. male gender 70.9%) from a study in Bahir Dar city, North West Ethiopia [17], the sample size generated with EPI INFO 7 for this factor was 568. Since the population size is < 10,000 using correction formula

- $n\mathrm{f}=\frac{n}{1+\frac{\mathrm{n}}{\mathrm{N}}}=\frac{568}{1+\frac{568}{1569}}$= 417 by taking 10% non-response rate
- nf =417 + 417* $$ \raisebox{1ex}{$10$}\!\left/ \!\raisebox{-1ex}{$100$}\right. $$ (non-response rate) =417 + 41.7 = ≈459

Therefore, the final sample size used was 459.

**Fig. 1:**
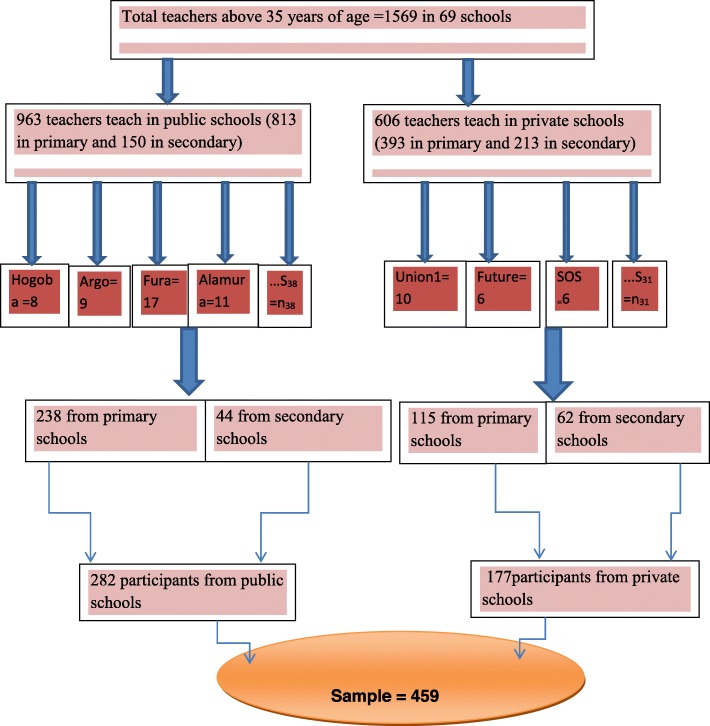
Schematic representation of the sampling procedure for the unmet need for presbyopia correction among school teachers in Hawassa city, South Ethiopia

A simple random sampling technique was used to select the samples proportionally from 69 schools. The number of teachers older than age 35 years in each school was identified by asking their current age using their name list. Using their name list as a sampling frame, 459 study participants were proportionally selected by the lottery method from all 69 schools. The selected participant who fulfilled the inclusion criteria (older than 35 years of age, whose near binocular VA worse than point 8 (N8) with best distance correction, and can improve to point 8 (N8) or better after refraction) was included in the study. The next study participant was selected using the lottery method if a selected individual didn’t fulfill the inclusion criteria (Fig. 1).

### Data collection tool and procedures

Data collection was done by trained 10 optometrists using a pre-tested structured questionnaire in Amharic version converted by a language expert, which ascertained information about socio-demographic data, associated factors, and a noninvasive ocular examination using a penlight, streak retinoscope, and full trial set and literate near acuity chart (N-chart). Distance visual acuity (unaided or habitual) was measured under the ambient light condition on the room corridor using Snellen’s E chart. Refraction with streak retinoscopy and subjective refinement was done for those with reduced unaided or habitual distance visual acuity (VA = 6/12 or worse in both measurements) and their near visual acuity was tested after best distance correction. Near visual acuity was directly measured for participants whose unaided or habitual distance visual acuity was 6/9 or better. Near visual acuity was measured for all selected individuals and for those with near visual acuity worse than N8 refraction was performed by adding + 0.25DS step by step until there is no difference with additional + 0.25DS lens. The final powers of the lens required to read the smallest line and near visual acuity were recorded. Two additional visits were conducted to get study participants who did not avail at the first visit.

### Operational definition

Presbyopia is defined as an inability to read N8 at 40 cm with best correction at distance, which can be corrected to N8 or better with near correction [19, 20].

Clinically significant presbyopia is defined as near visual acuity correction of ≥ + 1.00D in addition to distance correction [19].

#### Unmet need for presbyopia correction

Number of subjects unable to see N8 binocularly unaided or with their existing near correction that can be corrected to N8 or better after refraction.

Awareness of presbyopia is positive (aware) if answered (yes) for the question; “have you ever heard of the age-related near vision difficulty called presbyopia**?”**

The degree of presbyopia refers to the extent of age-related near vision difficulty due to a decline in the amplitude of accommodation of the eye, based on presenting unaided near visual acuity at 40 cm. Accordingly, presbyopia was classified as mild (near VA worse than N8 to N10), moderate (near VA worse than N10 to N18), and severe (near VA worse than N18) [19].

### Data processing and statistical analysis

After coding, cleaning, and checking for completeness, the data was entered into EPI INFO version 7 and exported to SPSS. The descriptive part of the data analysis was summarized using frequency distribution and means. Binary logistic regression analysis was performed to identify factors associated with unmet need for presbyopia correction. Since the number of variables was small, all variables were entered into the multivariable analysis. Enter variable selection method was used to inter variables to multivariable binary logistic regression analysis. Odds ratios (OR) with 95% confidence interval was used to assess the strength of association. A *P*-value of < 0.05 was considered a statistically significant association. Multi-colinearity was checked using variance inflation factor and goodness of fit was diagnosed with Hosmer and Lemeshow model fitness test. Hosmer and Lemeshow with a P-value of > 0.05 was considered as good model fitness.

### Ethical consideration

Before conducting the study, ethical clearance was obtained from School of Medicine, Ethical review committee, University of Gondar. An official letter was obtained from Gondar University College of Medicine and the Health Sciences Department of Optometry and Hawassa City Health Bureau. Also, oral permission was obtained from each school director after a brief explanation of the purpose of the study for school directors. Written informed consent was obtained from each study participant after explaining the aim of the study to proceed to the examination and interview step. The confidentiality of the data was ensured and the consent declared that participants’ participation is voluntary. They were also informed that there is no any risk through participating in this survey. It was also clarified that they have full right to refuse from participating in the study and to withdraw at any time they wish. They would also have a full right to contact and ask the authors whatever they want.

## Results

### Socio-demographic characteristics of the study participants

Four hundred fifty-nine study participants were included in the study with a response rate of 95.21%. The mean age of participants was 47.85 years (± 7.33SD). More than half of the study participants (51.03%) were female and about two-third of study participants (67.28%) were first degree holders (Table 1).

**Table 1:** Socio-demographic characteristics and unmet need for presbyopia correction among school teachers in Hawassa city, South Ethiopia (*n* = 437)

Variables	Frequency n (%)	Uncorrected presbyopia
		Frequency n (%)
Age in years		
36–45	212 (48.51)	167 (78.77)
46–55	132 (30.21)	42 (31.82)
> 56	93 (21.28)	15 (16.13)
Sex		
Male	214 (48.97)	94 (43.93)
Female	223 (51.03)	130 (58.30)
Educational status		
Diploma	111 (25.40)	88 (79.28)
Degree	294 (67.28)	125 (42.52)
MSc/MA	32 (7.32)	11 (34.34)
Marital status		
Married	385 (88.10)	198 (51.43)
Single	52 (11.90)	26 (50.00)
^a^Income per month in ETB		
2748–5250	221 (50.57)	157 (71.04)
5251–7800	153 (35.01)	55 (35.95)
> 7800	63 (14.42)	12 (19.05)
Type of school		
Public	272 (62.24)	122 (44.85)
Private	165 (37.74)	102 (61.82)
Level of school		
Primary	352 (80.55)	191 (54.26)
Secondary	85 (19.45)	33 (38.82)

^a^income is categorized using the 2018 Ethiopian taxation rate

### The proportion of unmet need for presbyopia correction

**Fig. 2:**
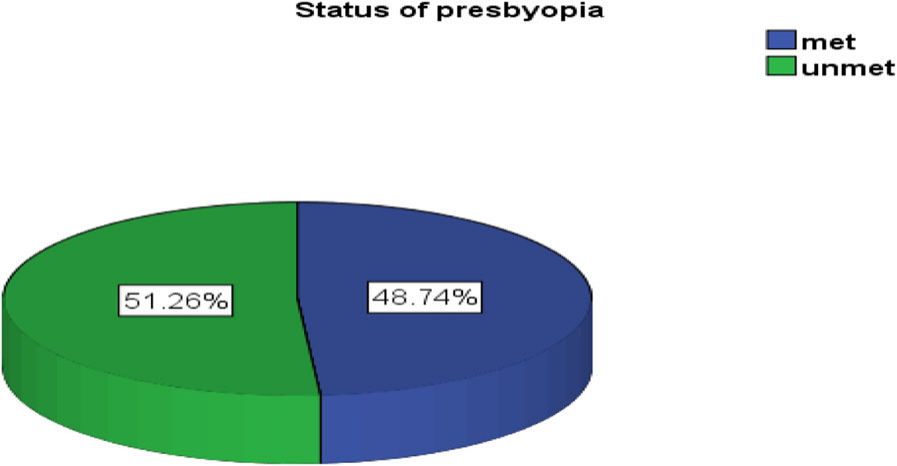
Proportion of unmet need for presbyopia correction among school teachers in Hawassa city, South Ethiopia

The proportion of unmet need for presbyopia correction was calculated using the formula: Unmet need for presbyopia correction = (No of individuals without presbyopia correction/No of individuals with presbyopia correction + without presbyopia correction)* 100%. Therefore, among 437 study participants with presbyopia, 51.26%(46.7–55.6%) had inadequate near correction, of which females accounting for more than half (Fig. 2).

The unmet need for presbyopia correction in those with a diploma, aged 36–45 years, and earning a monthly income of (2748–5250 ETB) was 79.28, 78.8, and 71.04% respectively (Table 1).

### Factors associated with unmet need for presbyopia correction

Fourteen variables as reflected in Table 2 including sex, age, educational status, marital status, monthly income, self-rating of current vision, degree of presbyopia, awareness of the presbyopia, history of the eye examination, family ocular history, type of school and school level were assessed with bivariable logistic regression analysis. Since the number of variables in this study was small, all variables were entered into a multivariable logistic regression to control for the effect of potential confounders. In multivariable analysis, being female (AOR 2.50; 95% CI: 1.51–4.15), age36–45 years (4.12; 95% CI 1.46–11.76), unaware of the presbyopia (AOR 2.36; 95% CI: 1.2–4.66), self-rating of current vision as good (AOR 3.5; 95% CI: 1.61–7.65) were significantly associated with unmet need for presbyopia correction (Table 2).

**Table 2:** Factors associated with the unmet need for presbyopia correction among school teachers in Hawassa city, South Ethiopia (*n* = 437)

Variables	Status of presbyopia			
	Unmet, n (%)(*n* = 224)	Met, n (%) (*n* = 213)	COR, (95% CI)	AOR,(95%CI)
Age				
36–45	167 (78.77%)	45 (21.23%)	19.3**(10.14–36.7)	4.12*(1.46–11.76)
46–55	42 (31.82%)	90 (68.18%)	2.43*(1.25–4.7)	0.96 (0.40–2.32)
> 56	15 (16.130%)	78 (83.87%)	1.00	1.00
Sex				
Female	130 (58.3%)	93 (41.7%)	1.78*(1.22–2.61)	2.50**(1.51–4.15)
Male	94 (43.9%)	120 (56.1%)	1.00	1.00
Educational status				
Diploma	88 (79.28%)	23 (20.72%)	7.3**(3.1–17.29)	0.59 (0.18–1.95)
Degree	125 (42.5%)	169 (57.5%)	1.41 (0.66–3.04)	0.39 (0.14–1.06)
MSc/MA	11 (34.38%)	21 (65.62%)	1.00	1.00
Marital status				
Single	26 (50%)	26 (50%)	0.94 (0.53–1.69)	1.05 (0.48–2.30)
Married	198 (51.43%)	187 (49.57%)	1.00	1.00
Monthly income in ETB				
2748–5250	157 (71.04%)	64 (29.96%)	10.43**(5.23–20.84)	0.86 (0.30–2.50)
5251–7800	55 (35.9%)	98 (64.1%)	2.39*(1.17–4.85)	1.96 (0.93–3.92)
> 7800	12 (19.04%)	51 (80.96%)	1.00	1.00
Type of school				
Private	122 (65.95%)	63 (34.05%)	1.99*(1.34–2.95)	0.68 (0.39–1.21)
Public	102 (40.48%)	150 (59.52%)	1.00	1.00
Level of school				
Primary	191 (54.26%)	161 (45.74%)	1.87*(1.15–3.03)	1.96 (0.93–3.92)
Secondary	33 (38.8%)	52 (61.2%)	1.00	1.00
Teaching stream				
Social science	109 (54.2%)	92 (45.8%)	1.24 (0.86–1.82)	0.92 (0.55–1.54)
Natural science	115 (48.7%)	121 (51.3%)	1.00	1.00
Awareness of presbyopia				
Unaware	152 (63.6%)	87 (36.4%)	3.06*8 (2.07–4.52%)	2.36*(1.2–4.66)
Aware	72 (36.4%)	126 (63.6)	1.00	1.00
Awareness of place for refraction				
Unaware	146 (63.2%)	85 (36.8%)	2.82**(1.91–4.16)	0.71 (0.36–1.42)
Aware	78 (37.9%)	128 (62.1%)	1.00	1.00
History of eye examination				
No	148 (71.5%)	59 (28.5%)	5.08**(3.38–7.65)	1.28 (0.71–2.30)
Yes	76 (33.04%)	154 (66.96%)	1.00	1.00
Family ocular history				
No	152 (69.7%)	66 (30.3%)	4.7*(3.14–7.0)	1.23 (0.65–2.32)
Yes	72 (32.9%)	147 (67.1%)	1.00	1.00
Self-rating of their current vision				
Good	41 (21.7%)	148 (78.3%)	10.16**(6.5–15.8)	3.5*(1.61–7.65)
Poor	183 (73.8%)	65 (26.2%)	1.00	1.00
Degree of presbyopia				
N10	48 (66.7%)	24 (33.3%)	11.88**(5.84–24)	1.37 (0.49–3.84)
N12-N18	159 (64.4%)	88 (35.6%)	10.74 (6.03–19.1)	2.28 (0.98–5.29)
Worse than N18	17 (14.4%)	101 (85.6%)	1.00	1.00

* = *P*-value < 0.05 ** = *p*-value < 0.001

Accordingly, individuals aged 36–45 years were 4.12 times more likely to have an unmet need for presbyopia correction compared with those 56 years and older. Being female was 2.50 times more likely to have an unmet need for presbyopia correction as compared to males. Individuals who were unaware of presbyopia were 2.36 times more likely to have an unmet need for presbyopia correction compared to their counterparts who were aware of it. Similarly, individuals who rate their current near vision as good despite having significant presbyopia were 3.5 times more likely to have an unmet need for presbyopia correction as compared to those who rate their current vision as poor.

## Discussion

The unmet need for presbyopia correction in Hawassa city, South Ethiopia was 51.26% (95% CI: 46.7–55.6%), which implies moderate priority according to the World Health Organization classification for presbyopia correction service provision. This proportion is lower than a report from Bahir Dar city, North West Ethiopia (69.2%) [17], Eretria (84.6%) [21], Kenya (93.7%) [1], Zanzibar (83.4%) [16] and Bangladesh (74.8%) [14]. The current study was conducted on school teachers whose near visual demand is high which may force them to have correction while the previous studies were community-based for whom detail near vision might be less essential. This difference may account for the variation of unmet need for presbyopia correction between those countries. The unmet need for presbyopia correction in this study was also, lower than studies from Nepal (90%) [22], Prakasam district, South India (56.8%) [11], Brazil (61%) [13] and Nicaragua (62.9%) [14]. The discrepancy in magnitude of unmet need of presbyopia correction might be due to the difference in study setting and residence.

The unmet need for presbyopia correction in this study was higher than reports from developing countries such as Ghana (29.6%) [4], Info Township- Nigeria (38.5%) [15], and Jakarta Indonesia (41.0%) [12]. In Ghana, teachers aged 40 years and above were included using the criterion of the distance of 30 cm to achieve at least N8 optotype. This age difference might account for the difference in the magnitude of uncorrected presbyopia compared to our study. In Nigeria teachers above 30 years were included which may account for the variation in unmet need for presbyopia correction between the current and the study in Nigeria. The burden difference between our study and Jakarta, Indonesia might be due to the presence of a school-based eye health program that includes visual acuity screening, refraction, and provision of free eyeglasses to students and teachers in Jakarta, Indonesia [12].

The unmet need for presbyopia correction in our study was similar to reports from Ido local government of Nigeria (54.5%) [18] and in Tanzania (46.5%) [23]. This might be due to similarities in sample size used, the mean age of study participants, and the criteria used in both studies.

This study identified that being in the age group 36–45 years, being female, unaware of presbyopia, self-rating of current near vision as good by the respondent were factors significantly associated with the unmet need for presbyopia correction.

Accordingly, being female was 2.50 times more likely to have an unmet need for presbyopia correction compared to males. This is similar to reports from Bahir Dar, North West Ethiopia [17], Info Township- Nigeria [24], and Timor-Leste [25]. Similarly, in Timor-Leste, unmet need for presbyopia correction was 79% for females while 68.8% in males. This is due to a higher prevalence and earlier onset of presbyopia in women due to physiological and physical reasons besides long life expectancy compared to men [5].

Individuals in the age group 36–45 years were almost 4 times more likely to have an unmet need for presbyopia correction compared to the age group 56 years and older. Age from 36 to 45 was similarly associated with the unmet need for presbyopia correction in studies done in Bahir Dar city, North West Ethiopia [17], and China [10]. This might be due to more near visual reduction in those 56 years and older necessitates them to have correction compared to the younger age group 36–45 years.

The current study found that the unmet need for presbyopia was nearly 2.36 times more in individuals who were unaware of presbyopia as compared to those aware of it. A previous study in Nigeria [15] and China [10], showed that lack of awareness of presbyopia was a barrier to obtaining presbyopia correction. In Nigeria, Info Township [15], (23.7%), and Ido local governments of Nigeria [18], (34.7%) were left uncorrected for presbyopia due to unaware of it. In China [10], 28.8% of subjects without correction reported a lack of being aware as a barrier. This might be since awareness of eye problems influences eye healthcare-seeking behavior. In the current study, 71% of study participants who were aware of presbyopia had a history of eye examination, which indicates that eye healthcare-seeking behavior is higher among individuals having awareness of presbyopia.

Individuals, who rate their current vision as good were 3.5 times more likely to have an unmet need for presbyopia correction compared to their counterpart rating as poor. In a previous study, in Indonesia [12], perception of their current vision as normal by the participants was reported as one of the barriers for seeking presbyopia correction. Most of the study participants were from primary schools where most of the teaching materials were written with large font size and picture compounded with increased computer use, mostly used at distance beyond 40 cm which minimizes near vision difficulty until a late stage of presbyopia which makes them left undetected and uncorrected.

## Conclusions

The unmet need for presbyopia correction among school teachers in the study area was ranked as a moderate priority area for intervention by WHO criteria for prioritization of presbyopia services. Being female, aged 36–45 years, lack of awareness about presbyopia and misperception of their current near visual status were factors affecting presbyopia correction. Since the factors affecting the unmet need for presbyopia correction identified in this study can be influenced by health education and vision screening, the Ethiopian federal ministry of health should facilitate eye health information dissemination concerning presbyopia through mass media. Also, the South Nations Nationalities Peoples State Health Bureau has to create linkage with different non-governmental organizations which work on eye-related activities to establish a school-based eye health program involving visual acuity screening, refraction, and spectacle provision. Similarly, eye health care professionals should provide health education on presbyopia and near visual impairment with a school-based refractive error correction campaign.

## Data Availability

The dataset on which the conclusion was made is available on request from Minychil Bantihun Munaw/minychilmedban@gmail.com or Balcha Negese/ balchanege@gmail.com

## Abbreviations

VA: Visual Acuity
N: Symbol represent point notation of near visual acuity chart
E- chart: Illiterate Tumbling Chart
EBT: Ethiopian Total Birr
MSc: Master of Science
MA: Master of Art
